# The effects of bar positioning, stance width, repetition, and load on muscle activity during the barbell back squat

**DOI:** 10.1371/journal.pone.0354893

**Published:** 2026-08-11

**Authors:** Chloe X. Y. Lee, Antony J. Crossman, Angela E. Kedgley

**Affiliations:** Department of Bioengineering, Imperial College London, London, United Kingdom; Università degli Studi di Milano: Universita degli Studi di Milano, ITALY

## Abstract

The barbell back squat is used widely across multiple sporting disciplines as part of strength and conditioning training. There are multiple training variations within performing a BBS. The hypothesis of this study was that bar positioning (high bar versus low bar), stance width (wide stance versus narrow stance), weight, and the number of repetitions completed (light weight – ten repetitions at 50% of maximum; heavy weight – three repetitions at 80% of maximum) can be manipulated for targeted training effects. The aim of the study was to quantify the effect squatting technique has on the activation of the rectus femoris, vastus medialis, vastus lateralis, biceps femoris, semitendinosus, gastrocnemius and gluteus maximus. Surface electromyography and kinematic data were recorded from eighteen healthy participants (twelve male, six female). Mean total area under the activation curve was compared for each muscle. Adopting a wide stance width showed increases in both peak and cumulative activation of the vastus medialis (p < 0.015). Squatting with increased weight showed increased cumulative activation of the vastus lateralis and semitendinosus (p < 0.041). An increased peak activation of the biceps femoris was observed when a high bar and wide stance technique was performed with a heavy weight compared to a light weight (p = 0.047). A narrow stance squat showed a 3.7° increase in peak knee flexion (p = 0.010) and a decrease of 8.2° of hip abduction (p < 0.001). A low bar position revealed increased hip flexion of 3.4° (p = 0.012). Overall, consistent muscle activations did not favour any combination of squat parameters. Athletes wishing to increase strength may choose one of several combinations of squat stance, bar positioning, and weight-to-repetition ratios, guided by what is most comfortable for the athlete.

## Introduction

The barbell back squat (BBS) is used widely in strength and conditioning training programs for its functional nature, which allows the overloading of many muscles in a single exercise [1]. Many muscle groups contribute to the execution of stable and controlled movement during the BBS. The muscles in the legs that have featured most frequently in previous studies of the BBS exercise are the rectus femoris, vastus medialis, vastus lateralis, biceps femoris, semitendinosus, gastrocnemius and gluteus maximus [1]. Variations in posture and technique are likely to result in alterations in muscle activation. Higher loading has been shown to result in greater muscle hypertrophy compared to lower loading, as long as thresholds for failure are not reached [2].

There are little data to support the choices of squat parameters by powerlifters and athletes. Athletes can adopt two bar positions on the back during the BBS, the first where the bar sits atop the trapezius muscles, in a high bar position, or lower on the back, at least 3 cm from the top of the anterior deltoids, in a low bar position [3]. The biomechanical effects of low bar BBS compared to high bar BBS has been estimated through musculoskeletal modelling, the results showed that a high bar barbell position generated a greater muscle activity in the quadriceps muscles, whereas the erector spinae, hip adductors and gluteal muscles had greater muscle activation generated during the low bar BBS [4].

The effect stance width has on the activation of the leg muscles during the BBS exercise has also been investigated [5]. In wide stance squats athletes had their feet positioned at twice their hip width. In narrow stance squats athletes had their feet at approximately one and a quarter times their hip width. It was found that there was a higher activation of the gluteus maximus when using a wide stance compared to a narrow stance, and a higher activation in the rectus femoris, vastus lateralis, and vastus medialis for higher weights compared to lower weights [5]. Similarly, a second study investigated stance width across 70 lifters performing squats at 70% of their one repetition maximum (1RM) [6]. It was found that narrow stance squats increased the forces on the quadriceps muscles and wide stance increased force in the gluteus maximus, biceps femoris, and semitendinosus [6].

Analysis of the effect that stance width and barbell position have on progressing the squat through the sticking region of the BBS found that training with a high bar position and a narrow stance width targeted knee extensor muscles whilst a low bar position and wide stance width focussed stress on the hip extensors [7]. The sticking region is an interesting point of research when assessing improving top end strength within the BBS. However, many sports use the BBS as part of a strength and conditioning training program where maximum efforts may be avoided to reduce fatigue from accessory exercises to the main sporting discipline. Therefore, the effect these parameters have on the entire range of motion should be investigated.

A comparison of muscle activation at the sticking point of participants when performing BBS at 90%, 100% and 102% of their 1RM found that there was reduced activity of hip extensor muscles when participants failed a squat [8]. The comparison was again assessing factors that affect top end BBS performance which is not always the target of all athletes performing the exercise. Therefore, the effect of the relative weight being lifted by athletes should be investigated as it is evidenced that an increased weight on the bar does not necessarily increase muscle activation. The increased risk for injury and fatigue associated with heavy lifting may be avoided by using a lighter weight which still may elicit a similar neuromuscular response.

Prior research addressing the BBS has often focused on investigation of squat variations in isolation, e.g., bar height or stance width, rather than combining two or more squat parameters. The present study sought to support or debunk common gym advice with a hypothesis that adaptations to squat technique and training method alters the distribution of muscle recruitment and thus affects training results for the athlete.

The aim of the study was to quantify the activation of the major muscle groups involved in the BBS while varying three parameters of the squatting technique. These parameters were bar positioning: high bar versus low bar; foot stance width: wide stance versus narrow stance; and weight-repetition ratio: high-weight, with a low number of repetitions versus low-weight, with a high number of repetitions. Bar positioning and foot stance are parameters chosen by the athlete and each athlete will likely have personal preferences for a particular stance and bar position. By providing an objective comparison of squat parameters, training plans may be better constructed to suit the personal goals of each individual.

The first hypothesis was that the low bar BBS would result in higher activation of the gluteus maximus compared to high bar BBS, which conversely would result in higher activation of the quadriceps, as predicted previously by musculoskeletal modelling [4]. The second hypothesis was that the biceps femoris, gluteus maximus and semitendinosus would exhibit higher activation during the wide stance squat and the rectus femoris, vastus medialis and vastus lateralis would exhibit higher activation during the narrow stance squats [5,6]. The third hypothesis was that the heavy weight squats would produce higher peak muscle activation compared to the light weight squats [2,5].

## Materials and methods

To test the hypotheses, a study was implemented in which bar position, stance width and relative weight on the bar each could be controlled as independent variables. In changing squatting technique, the body must adapt to different positioning of the weight on the back and the feet on the floor. Adapting to these changes can be measured in changes to kinematics and adaptations in muscle recruitment for the different motions. To assess the differences between the squatting variations, kinematics about the hip, knee and ankle and muscle activation of the rectus femoris, vastus medialis, vastus lateralis, biceps femoris, semitendinosus, gastrocnemius and gluteus maximus were all measured as dependant variables.

### Participants

Eighteen participants – twelve males (age: 23.0 years ± 2.0 years, height: 1.80 m ± 0.08 m, body mass: 83.0 kg ± 8.5 kg) and six females (age: 22.7 years ± 1.9 years, height: 1.67 m ± 0.07 m, body mass: 63.0 kg ± 4.5 kg) – volunteered to participate in this study. Of the eighteen participants, nine were powerlifters, and one was an Olympic style weightlifter, with the other participants recreationally performing strength training with free-weights. Six of the participants (n = 6, 33%) had competed nationally in their respective sports.

The study was approved by Imperial College London’s Science Engineering Technology Research Ethics Committee (reference number: 6445702). Recruitment took place between 10^th^ March 2023 and 30^th^ June 2023. All participants were asked to read an information sheet and sign a consent form. Participants completed a pre-screening questionnaire to ensure they had regular experience of the BBS. Prospective participants were excluded if they were pregnant, had sustained a musculoskeletal injury or had musculoskeletal surgery within the six months prior to participating in the study.

### Procedures

On passing the pre-screening, participants were invited to participate in the study. The study was split over two sessions with a rest day in between to balance participant rest and data collection efficiency [9]. Participants wore the same footwear across both days of data collection.

**Fig 1:**
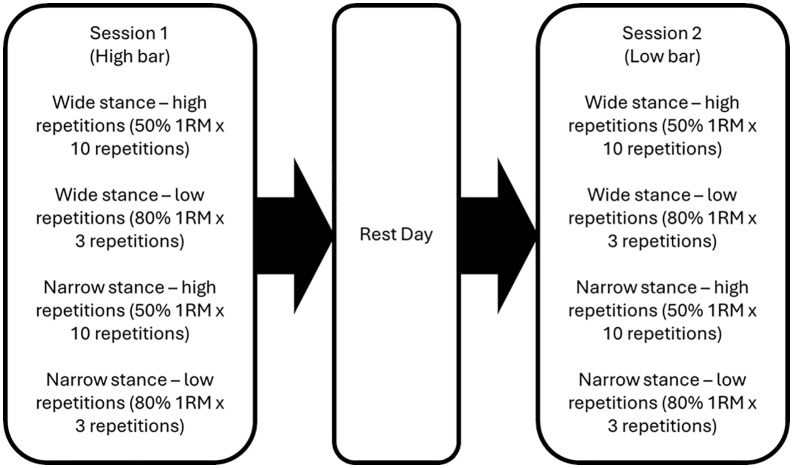
Study protocol across the two testing sessions. Weights were determined as a percent of each participant’s self-reported unassisted (without the use of assistive equipment, including belts, knee sleeves, or knee wraps) maximum weight they could lift for a single repetition of a full squat (1RM).

The first session included all high bar variations, and the second session included all low bar variations. At each session the participant completed squats with wide stance and narrow stance at heavy weight and light weight (Fig 1). Participants were asked to stand with their feet spaced approximately one and a quarter times hip width apart for the narrow stance squats and twice their hip width for wide stance squats [5]. Both stances were verified visually and approved during the participant’s warm-up squats. The achieved depths of the squats were also assessed visually during the trials with the research team ensuring competition depth was achieved for each squat. Participants were instructed to squat at their own cadence that was necessary to successfully perform the exercise.

The weight each participant lifted was determined relative to their self-reported unassisted maximum for a single repetition (1RM), which was the maximum weight the participant could lift for one full squat without the use of assistive equipment, including belts, knee sleeves, or knee wraps (males: 150.0 kg ± 37.8 kg, females: 60.0 kg ± 21.9 kg). Heavy weight squats were defined as 80% of a participant’s 1RM [10] and had a low number of repetitions, which was chosen as three, in line with previous studies [10,11]. Low weight squats were defined as 50% of a participant’s 1RM [10], with ten repetitions [11]. These weight and repetition combinations were chosen to protect the participants as two heavy weight sets were required per session, meaning that testing at 100% 1RM would not be feasible or safe for the participants as it would have carried a high injury risk.

**Fig 2:**
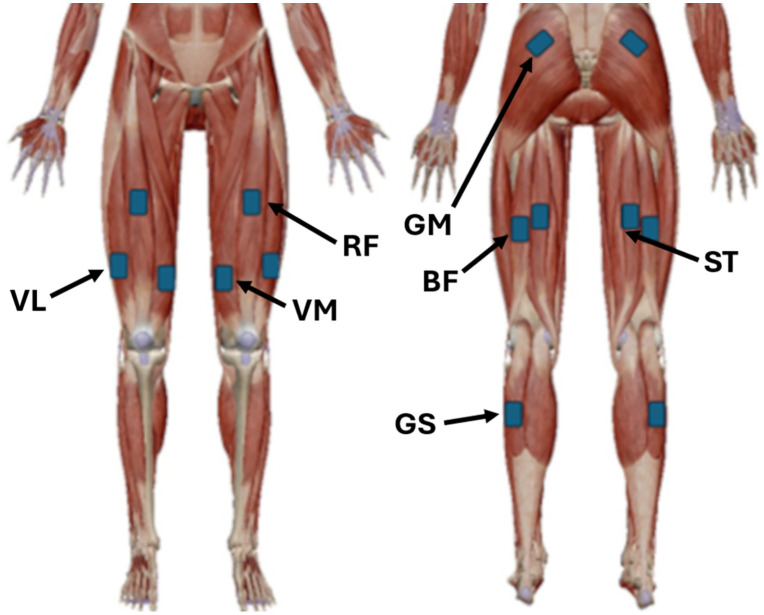
Placement of surface electromyography sensors on rectus femoris (RF), vastus medialis (VM), vastus lateralis (VL), gluteus maximus (GM), biceps femoris (BF), semitendinosus (ST), and gastrocnemius (GS), created based on images from Visible Body [Version 2018.5, Visible Body, Framingham, USA].

Fourteen surface electromyography (sEMG) sensors [Trigno, Delsys, Natick, MA, USA] were placed on the lower limbs of the participants (Fig 2). Eight Trigno sensors (27 mm x 46 mm x 13 mm) were placed on the rectus femoris, vastus medialis, vastus lateralis, and biceps femoris of the left and right legs. Six Trigno Mini sensors were used (25 mm x 12 mm x 7 mm) on the semitendinosus, gastrocnemius, and gluteus maximus of the left and right legs. The electrode bodies had 1 x 5 mm 99.9% silver parallel bars with 10 mm inter-electrode spacing. Alcohol wipes were used to clean the surface of the skin to allow proper adhesion of the sensors on the skin. The EMG sensors were placed on the main body of the muscles, identified through palpation, and aligned with the muscle fibres. sEMG data were captured using EMGWorks Acquisition [version 4.7.8, Delsys, Natick, MA, USA], exported using EMGWorks Analysis [Delsys, Natick, MA, USA] and analysed using MATLAB [version 2019 A, Mathworks, Natick, MA, USA]. The sEMGs output values of voltage magnitude corresponded to the muscle activity, with a higher voltage indicating more activity.

To measure and record the joint kinematics during the squats, OpenCap (OpenCap, version 4.1, SimTK, Palo Alto, CA, USA), a marker-less motion tracking software, was used [12]. OpenCap was selected over marker-based systems due to its portability into a gym environment. Additionally, OpenCap is not affected by reflective surfaces, that are commonplace in gyms, and which would create many phantom markers in a retroreflective marker-based system.

Participants were instructed to warm up for the squatting exercises using their usual routine, building up to the weights being recorded. The rest breaks between each set of squats were set by the participant with no restriction on duration. The participants were able to use self-prescribed warm-ups, self-selected intermediate squat weights, and rest breaks to reduce the risk of injury to the participant. At the end of each session, participants were given time and space to cool down with their usual routine to minimise the risk of injury or delayed onset muscle soreness.

sEMG data from the squat trials were filtered using bandpass filters (Table 1) [13,14]. Muscle activation data were normalised using the maximum activation across all trials of each muscle on each day of participation [15]. From the normalised squat data, for each muscle in each set, the mean peak contractile EMG value was found by calculating the mean of the maximum value of each repetition. The mean total area under the sEMG plot of voltage against time for each squat was also calculated.

**Table 1:** Muscles and their corresponding cut-off frequencies.

Muscle(s)	High-Pass Filter [Hz]	Low-Pass Filter [Hz]
Rectus femoris, vastus medialis, vastus lateralis, biceps femoris, semitendinosus	20	500
Gastrocnemius	20	450
Gluteus maximus	10	500

The 3D virtual marker coordinates were extracted from the data recorded by OpenCap and analysed using MATLAB. A segment coordinate system was defined for each of the segments in the lower limb [16]. A ZXY axis rotation sequence was used to calculate joint angles for the hips and knees, with rotations representing flexion/extension, adduction/abduction and internal/external rotation, respectively. Although all ranges of motion were calculated, only functionally trainable motions were analysed. Therefore, the flexion/extension of the hip and knee joint and abduction and adduction of the hip were taken forward. The maximum and minimum joint angles, and the ranges of rotations were found for each of the combinations of squat parameters. The joint angles were used to assist in the interpretation of the sEMG data.

### Statistical analysis

The EMG data were statistically analysed, using Shapiro-Wilk tests for normality, followed by a repeated measures ANOVA test where stance width, bar position, weight lifted and limb were tested as factors (significance was defined as p < 0.05) to determine if there were any differences between limbs. As no difference was found between limbs, the mean of the paired muscles in each limb were found and the repeated measure ANOVA retested with stance width, bar position and weight lifted included as factors. This was done based on the priori power analysis [17], where a sample size of 16 was required with an alpha of 0.05 and a power of 0.80.

The kinematic data were statistically analysed, using Shapiro-Wilk tests for normality, followed by a three-way repeated measures ANOVA test where stance width, bar position and weight lifted were tested as factors (significance was defined as p < 0.05) to determine differences between squat parameters. 11 of the 144 squat trial recordings were rejected due to obvious inaccuracies in the kinematic tracking. However, the EMG data for these recordings were retained due to the visual assessment of the squatting technique made by the investigators at the time of data collection.

## Results

**Fig 3:**
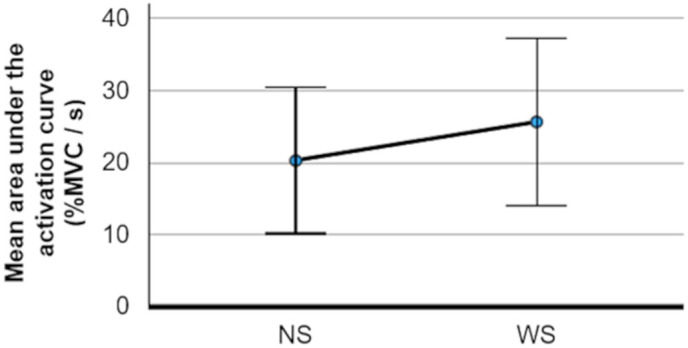
Mean area under the vastus medialis muscle activation curve (with 95% confidence interval) normalised to the maximum activation within each session (%MVC) comparing all narrow stance (NS) and all wide stance (WS) squatting variations.

A main effect of stance was observed for the vastus medialis for both the area under the activation curve (p = 0.006, F = 20.625, η^2^p = 0.805) and peak activation (p = 0.015, F = 13.415, η^2^p = 0.728). The average area under the curve when performing wide stance squats showed a relative increase of 26.6% when compared to narrow stance variations (Fig 3). The peak activation also showed a relative increase of 37.0% when compared to performing wide stance squats compared to narrow stance variations.

**Fig 4:**
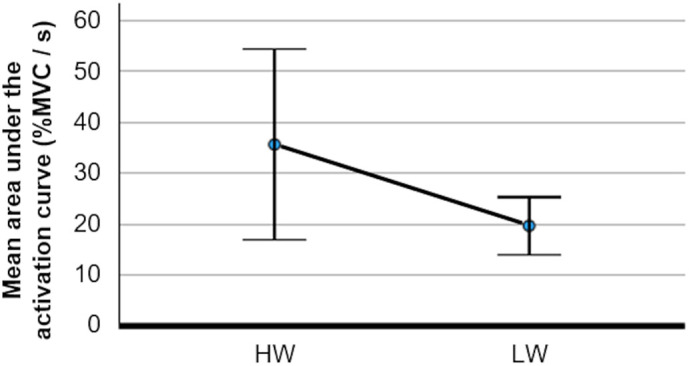
Mean area under the vastus lateralis muscle activation curve (with 95% confidence interval) normalised to the maximum activation within each session (%MVC) comparing all heavy weight (HW) and all light weight (LW) squatting variations.

A main effect of weight was observed for the vastus lateralis for the area under the activation curve (p = 0.041, F = 7.522, η^2^p = 0.601), revealing an average relative increase of 80.7% when squatting with a heavy weight and low repetitions compared to a light weight with high repetitions (Fig 4).

**Fig 5:**
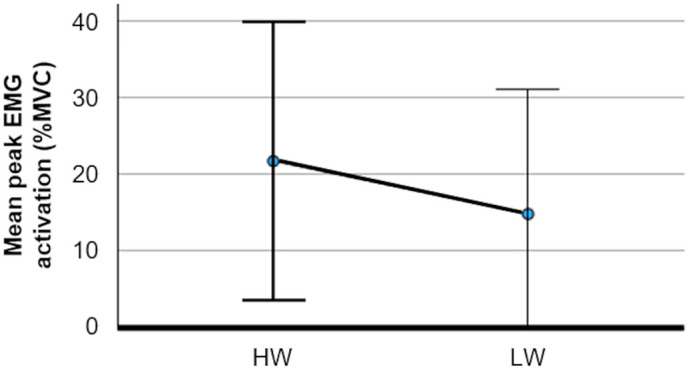
Mean peak activation of the semitendinosus muscle activation (with 95% confidence interval) normalised to the maximum activation within each session (%MVC) comparing all heavy weight (HW) and all light weight (LW) squatting variations.

A main effect of weight was observed for the semitendinosus for the peak activation (p = 0.011, F = 15.375, η^2^p = 0.755), revealing an average relative increase of 46.5% when squatting with a heavy weight and low repetitions compared to a light weight with high repetitions (Fig 5).

A three-way interaction was observed for the peak activation of the biceps femoris (p = 0.041, F = 11.847, η^2^p = 0.798). A Bonferroni-adjusted post-hoc pairwise comparison revealed a 95.8% relative increase for trials adopting a high bar position, wide foot stance and heavy weight and low repetition compared to a low weight and high repetition range (p = 0.047).

No effects were present in the rectus femoris, gastrocnemius or gluteus maximus.

A main effect of stance width was found for flexion/extension of the knee (p = 0.010, F = 12.493, η^2^p = 0.641), revealing that adopting a narrow stance width increased knee flexion by 4.7°.

A main effect of bar position was found for flexion/extension of the hip (p = 0.012, F = 11.48, η^2^p = 0.621), revealing that adopting a low bar position increased hip flexion by 3.4°.

**Fig 6:**
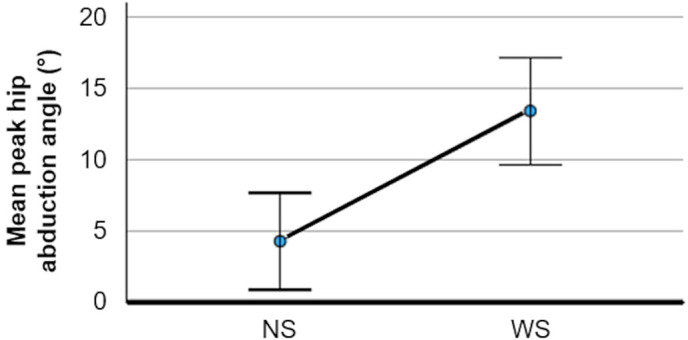
Mean peak hip abduction (with 95% confidence interval) comparing all narrow stance (NS) to all wide stance (WS) squatting variations.

A main effect of stance width was found for abduction/add of the hip (p < 0.001, F = 172.895, η^2^p = 0.910), revealing that adopting a wide stance width increased hip abduction by 8.2° (Fig 6).

## Discussion

The effects of combinations of variations in bar positioning, foot stance, and weight-repetition ratio during BBS have not previously been documented. The study aimed to determine if activation patterns of the major muscle groups involved in the BBS can be manipulated through varying three parameters of the squatting technique: bar-positioning, foot stance, and weight-repetition ratio. This information fills a gap in research around BBS stance and training style, thus allowing individuals to make informed decisions for their training, tailored to their own strengths and goals.

In total, there were five combinations that showed differences in muscle activation (p < 0.05). There were no main effects or interactions which supported the first hypothesis that low bar variations of squatting elicit greater activation in the gluteus maximus and that narrow stance variations elicit higher activation in the quadriceps muscles. Therefore, the results of this study did not agree with the findings of Glassbrook et al., who found that high bar BBS had higher forces on the quadriceps muscles compared to the low bar BBS [4]. This may be due to the convolution of the weight lifted and the number of repetitions with the other parameters, creating different squat conditions from those tested in prior studies.

Hypothesis two was partially disproven as the vastus medialis achieved higher peak activation when a wide stance was adopted as opposed to the expected narrow stance. The function of the vastus medialis is to extend the knee and stabilize the patella. A possible reason for the increase in the activation of this most medial quadriceps muscle is to maintain patella alignment during knee flexion whilst the hip is in an abducted position, evidenced by the kinematics findings of this research. It is known that weakness of the hip may result in patellofemoral pain [18] and so there is an effect of hip position on patella kinematics, affecting muscle forces to maintain position.

The third hypothesis linking increased weight on the bar to increased peak muscle activation was proven true in the vastus lateralis, semitendinosus and biceps femoris. This supports existing studies that showed that heavy weight compared to light weight strength training would result in overall higher muscle activation [2,5]. However, a total consensus was not proven across all muscles.

This study had a participant pool of varying lifting abilities and experience levels, from recreational gym-goers to competitive powerlifters and Olympic style weightlifters. This variation means the results are not biased depending on the build or preferences of an individual based on their sport, existing training style, or experience. Both male and female volunteers participated and, again, spanned the various squatting experience levels.

Many sports that include the BBS, or a similar motion, like powerlifting and weightlifting, allow for assistive equipment. This includes items such as lifting belts, wrist wraps, knee sleeves and squat suits. All this equipment aims to provide increased stability and support throughout the squatting motion, which is likely to allow the individual to lift more weight safely. In the future, the effect of incorporating assistive equipment on muscle activation could be investigated, as it may result in participants having a higher 1RM. Additionally, a similar method and study flow could be used for other exercises, such as the bench press and deadlift.

One substantial limitation to the protocol used in this study is that data were collected over two days. This was implemented so as not to fatigue the participants and risk injury during the study. The limitation of separate testing days is the potential for participants to not feel as strong or mobile across two different days, resulting in different performance levels. This was mitigated by giving participants one rest day between data collection sessions, which allowed the participants to recover whilst maintaining similar body state between days. Additionally, testing on two separate days means variable EMG electrode placement between testing sessions. To mitigate this, EMG data were normalised separately for each day of testing relative to the maximum achieved contraction of each muscle, thus establishing independent baseline comparators for the EMG data acquired each day. No muscle recruitment differences were noted due to bar position in this study. Future studies adopting a similar protocol should randomise the bar position between the two days.

Another limitation was the use of markerless motion capture. The OpenCap software was not trained on BBS recordings, meaning that the automatic identification and tracking of landmarks was not optimised for the BBS movement. However, the system has been validated, with optical motion capture as the gold standard, for bodyweight squatting, yielding an accuracy of 4.5° [12]. As this study only focussed on lower limb kinematics, any errors in kinematics of the upper limbs due to the barbell did not affect the results.

Performing squats with a wide stance and at a heavy relative weight to the participants capabilities increased some muscle activations in the legs. However, more generally, no singular combination of the squat parameters can be favoured for its increased activation of a muscle group. Instead, athletes should choose the method that feels best for them, focussing on safety and stability to reduce injury risk, as opposed to forcing less natural motion, with the objective of eliciting specific results.

## Conclusion

Overall, few effects of squatting variations were noted in the neuromuscular response of muscles. Adopting a wide stance and using a heavier weight may illicit an increase in muscle activation of both knee and hip extensors. However, no other consistent change in muscle activity was noted across squatting variations, meaning that the BBS is a good compound movement activating all muscles of the lower limb. Technique and repetition ranges should be selected for comfort of the athlete, as opposed to targeting specific results, as all methods activate the muscles in the legs.

## Supporting information

S1 File Lee PLoS ONE – Digital supplementary content. (Worksheet 1 – EMG Data) Mean area and mean peak for rectus femoris, vastus medialis, vastus lateralis, biceps femoris, semitendinosus, gastrocnemius, and gluteus maximus for right and left limbs for all squat variations. (Worksheet 2 – Hip FlexExt) Hip flexion-extension angle for all squat variations. (Worksheet 3 – Hip AbdAdd) Hip abduction-adduction angle for all squat variations. (Worksheet 4 – Knee FlexExt) Knee flexion-extension angle for all squat variations. All data are presented for 18 participants.
(XLSX)
